# Optimization of Cellulase Production from Bacteria Isolated from Soil

**DOI:** 10.5402/2013/985685

**Published:** 2013-02-19

**Authors:** Sonia Sethi, Aparna Datta, B. Lal Gupta, Saksham Gupta

**Affiliations:** Department of Biotechnology, Dr. B. Lal Institute of Biotechnology, Malviya Industrial Area, Malviya Nagar, Jaipur 302017, India

## Abstract

Cellulase-producing bacteria were isolated from soil and identified as *Pseudomonas fluorescens*, *Bacillus subtilIs*, *E. coli*, and *Serratia marcescens*. Optimization of the fermentation medium for maximum cellulase production was carried out. The culture conditions like pH, temperature, carbon sources, and nitrogen sources were optimized. The optimum conditions found for cellulase production were 40°C at pH 10 with glucose as carbon source and ammonium sulphate as nitrogen source, and coconut cake stimulates the production of cellulase. Among bacteria,* Pseudomonas fluorescens* is the best cellulase producer among the four followed by *Bacillus subtilis*, *E. coli*, and *Serratia marscens*.

## 1. Introduction

Cellulose is the most abundant biomass on Earth [1]. It is the primary product of photosynthesis in terrestrial environments and the most abundant renewable bioresource produced in the biosphere [2, 3]. Cellulose is commonly degraded by an enzyme called cellulase. This enzyme is produced by several microorganisms, commonly by bacteria and fungi [4–7]. 

Cellulose is the principal constituent of the cell wall of most terrestrial plants. The source of cellulose is in plants and it is found as microfibrils (“2–20 nm” in diameter and “100–40,000 nm” long). These form the structurally strong framework in the cell walls. Despite a worldwide and enormous utilization of natural cellulosic sources, there are still abundant quantities of cellulosic sources and there are still abundant quantities of cellulose containing raw materials and waste products that are not exploited or which could be used more efficiently. The problem in this respect is, however, to develop processes that are economically profitable. Complete hydrolysis of the enzyme requires synergistic action of 3 types of enzymes, namely, cellobiohydrolase, endoglucanase or carboxymethylcellulase (CMCase), and beta-glucosidases [8].

Bacteria which have high growth rate as compared to fungi have good potential to be used in cellulase production. However, the application of bacteria in producing cellulase is not widely used. The cellulolytic property of some bacterial genera such as *Cellulomonas, Cellvibrio, Pseudomonas* sp [9]. *Bacillus,* and *Micrococcus* [7], was also reported. Enzyme production is closely controlled in microorganisms and for improving its productivity these controls can be ameliorated. Cellulase yields appear to depend upon a complex relationship involving a variety of factors like inoculums size, pH value, temperature, presence of inducers, medium additives, aeration, growth time, and so forth [7]. 

Enormous amounts of agricultural, industrial, and municipal cellulosic wastes have been accumulating or used inefficiently due to the high cost of their utilization processes [10]. Cellulose, a polymer of glucose residues connected by beta 1,4 linkages, being the primary structural material of plant cell wall, is the most abundant carbohydrate in nature [11]. Therefore, it has become of considerable economic interest to develop processes for effective treatment and utilization of cellulosic wastes as inexpensive carbon sources. Cellulase is the enzyme that hydrolyses the beta 1,4 glycosidic bonds in the polymer to release glucose units [12].

 Cellulose containing wastes may be agricultural, urban, or industrial in origin; sewage sludge might also be considered a source of cellulose since its cellulosic content provides the carbon needed for methane production in the anaerobic digestion of sludge. Agricultural wastes include crop residue, animal excreta and crop-processing wastes, slashing generated in logging, saw dust formed in timber production, and wood products in forestry originated activities. The previous negative attitude in which wastes were viewed self-consciously as valueless and even offensive and for disposal only has been replaced in large part by a positive view in which wastes are recognized as raw materials of potential value [13]. 

This cellulose-degrading enzyme can be used, for example, in the formation of washing powders, extraction of fruit and vegetable juices, and starch processing [14]. Cellulase is produced by a large number of microorganisms. They are either cell bound or extracellular. Although a large number of microorganisms can degrade cellulose, only a few of them produce significant quantities of free enzymes capable of completely hydrolysing crystalline cellulose [15].

 Cellulases are used in the textile industry for cotton softening and denim finishing; in laundry detergents for colour care, cleaning; in the food industry for mashing; in the pulp and paper industries for drainage improvement and fibre modification, and they are even used for pharmaceutical applications [16]. In nutshell, the cellulose enzymes will be commonly used in many industrial applications and the demands for more stable, highly active and specific enzymes will also grow rapidly. So, cellulose enzyme will be the most stirring technology of future. And continuous research for advances in speckled aspects for cellulose production (such as cost, substrate specificity, and specific activity) is desired to achieve improved technoeconomic feasibility.  The present work was carried out to optimize the nutritional and environmental parameters for improving cellulose production by bacterial strains. 

## 2. Experimental

### 2.1. Screening and Isolation of Bacteria

Cellulase-producing bacteria were isolated from soils by the dilution pour plate or spread plate method using CMC agar media. The plates were incubated at 45, 50, and 55°C for 24 hours. To visualize the hydrolysis zone, the plates were flooded with an aqueous solution of 0.1% Congo red for 15 min and washed with 1 M NaCl [17]. To indicate the cellulose activity of the organisms, diameter of the clear zone around colonies on CMC agar was measured. Besides, a more quantitative assay method was used to determine the cellulose activity of the selected bacterial isolate in liquid medium. The cellulase activity of each culture was measured by determining the amount of reducing sugars liberated by using a DNS method [18]. A bacterial isolate with the highest activity was selected for optimization of cellulose production.

### 2.2. Bacterial Identification

The bacterial isolates were presumptively identified by means of morphological examination and some biochemical characterizations. The parameters investigated included colonial morphology, gram reactions, endospore formation, catalase production, VP reaction, indole production, starch hydrolysis, citrate utilization, and gelatine hydrolysis. The results were compared with Bergey's Manual of Determinative Bacteria [19]. 

### 2.3. Enzyme Production Medium

Production medium contained (g/L) glucose 0.5 gm, peptone 0.75 gm, FeSO_4_ 0.01 gm, KH_2_PO_4_ 0.5 gm, and MgSO_4_ 0.5 gm. Ten millilitres of medium were taken in a 100 mL conical flask. The flasks were sterilized in autoclave at 121°C for 15 min, and after cooling, the flask was inoculated with overnight grown bacterial culture. The inoculated medium was incubated at 37°C in shaker incubator for 24 h. At the end of the fermentation period, the culture medium was centrifuged at 5000 rpm for 15 min to obtain the crude extract, which served as enzyme source.

### 2.4. Enzyme Assay

Cellulase activity was measured following the method of Miller [18]. Briefly, a reaction mixture composed of 0.2 mL of crude enzyme solution plus 1.8 mL of 0.5% carboxymethyl cellulose (CMC) in 50 mM sodium phosphate buffer (pH 7) was incubated at 37°C in a shaking water bath for 30 min. The reaction was terminated by adding 3 mL of DNS reagent. The colour was then developed by boiling the mixture for 5 min. OD of samples was measured at 575 nm against a blank containing all the reagents minus the crude enzyme. 

### 2.5. Process Optimization for Maximum Cellulase Production

#### 2.5.1. pH

Flasks with broth containing the optimum concentration of substrate and carbon source are taken and the pH of the broth is adjusted to 7.0, 8.0, 9.0, 10.0, and 11.0 in different flasks using 1 N HCl and 1 N NaOH and sterilized. The cultures are inoculated and incubated at particular temperature. At the end of incubation period, the cell-free culture filtrate is obtained and used as enzyme source.

#### 2.5.2. Temperature

Production medium at pH 7 was inoculated with overnight grown selected bacterial strain. The broth was incubated at different temperatures from 35, 40, 45, 50, 55, and 60°C for 24 h. At the end of incubation period, the cell-free culture filtrate is obtained and used as enzyme source.

#### 2.5.3. Carbon Sources

The effect of various carbon sources such as starch, glucose, maltose, lactose, and fructose at the concentration of 1 to 5% was examined in the production medium.

#### 2.5.4. Nitrogen Sources

Various nitrogen sources like yeast extract, peptone, urea, and ammonium sulphate were examined for their effect on enzyme production by replacing 0.5% peptone in the production medium.

#### 2.5.5. Agro-Based Waste Material

To find out the suitability of agro-based waste as substrate for enzyme production, different substrates, that is, groundnut cake, coconut cake, soy cake, and wheat bran, are taken in the growth medium under submerged condition. The enzyme activity is measured after 24 h for enzyme production.

## 3. Results and Discussion

Cellulase-producing bacteria were isolated from soil. Based on the morphological and biochemical characteristics, the isolates were identified as *Pseudomonas fluorescens, Bacillus subtilus, E. coli, *and *Serratia marscens*.

### 3.1. Effect of pH

**Figure 1:**
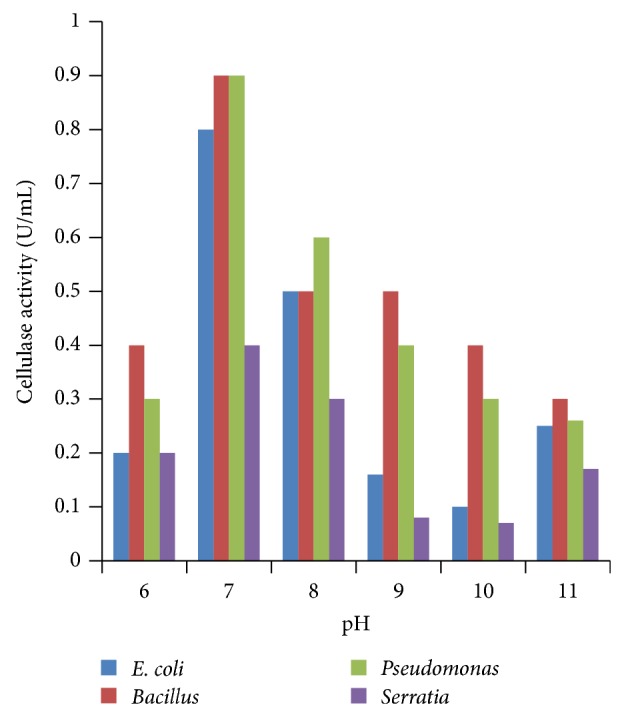
Effect of pH on cellulase activity.

All the four isolates were allowed to grow in media of different pH ranging from 6.0 to 11.0. Maximum enzyme activity was observed in medium of pH 9.0–11.0 in case of *E. coli*, *Pseudomonas fluorescens, Bacillus subtilis,* and *Serratia marscens *(Figure 1). This result was in correlation with the finding of other workers for different *Bacillus subtilis* strains [20–22].

### 3.2. Effect of Incubation Temperature

**Figure 2:**
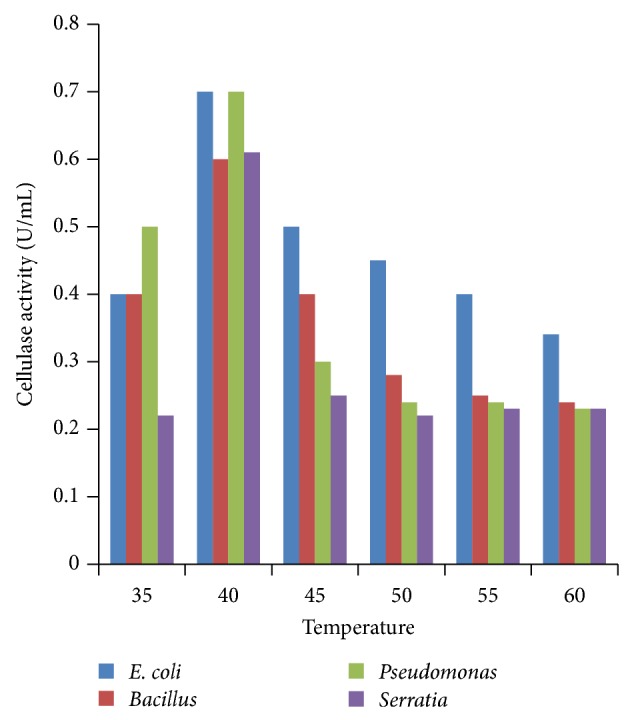
Effect of temperature on cellulase activity.

Enzyme activity recorded at different temperatures revealed that all the four bacteria yielded maximum cellulase production at 40°C (Figure 2). The temperature was found to influence extracellular enzyme secretion, possibly by changing the physical properties of the cell membrane. Optimum temperature for maximum growth of *Bacillus subtilis* 115 and *Bacillus subtilis *was 40°C [23]. These results are close those of Bakare et al. (2005) [24] who found that the cellulase enzyme produced by *Pseudomonas fluorescence *was activated at 30 to 35°C showing the optimum temperature at 35°C. Ray et al. [25] reported that minimum cellulase yield was observed when fermentation was carried out at 45°C, while maximum yield was obtained at 40°C by *Bacillus subtilis *and *Bacillus circulans*. Immanuel et al. [7] also recorded maximum endoglucanase activity in *Cellulomonas*, *Bacillus, *and *Micrococcus *sp. at 40°C and neutral pH.

### 3.3. Effect of Carbon Source

**Figure 3:**
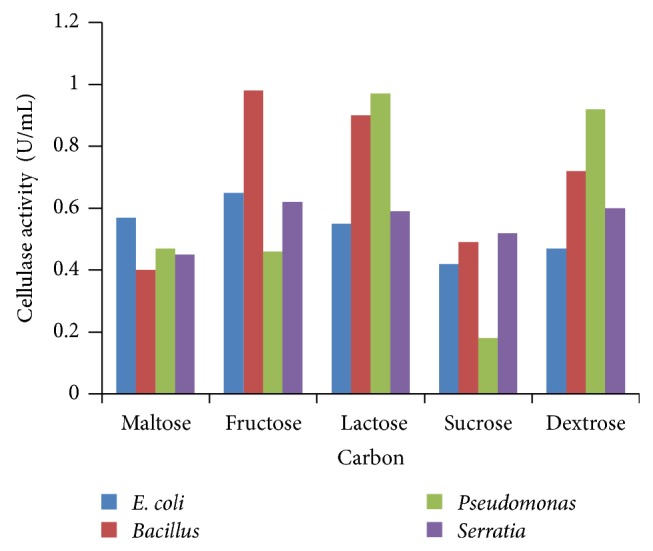
Effect of carbon sources.

Various sources of carbon such as starch, fructose, maltose, and sucrose were used to replace glucose which was the original carbon source in growth media. Results obtained showed that glucose brought the highest cellulase production compared to other carbon sources at 24 h incubation. Lactose and fructose also showed high cellulase production at 24 h of incubation. Hence, glucose was found to be the best source for cellulase production (Figure 3). Glycerol is the best substrate for cellulase production with the efficiency of 28.7% on the added substrate weight. Ishihara et al. [26] studied the utilization of D-xylose as carbon source for the production of cellulase membrane [27] and deduced that xylose is not well metabolized by any bacterial strains that exhibited high cellulose production in glucose medium, whereas sucrose, glucose, and mannitol were found to be suitable for optimum levels of cellulase production [28]. 

### 3.4. Effect of Different Concentrations of Carbon Sources

**Figure 4:**
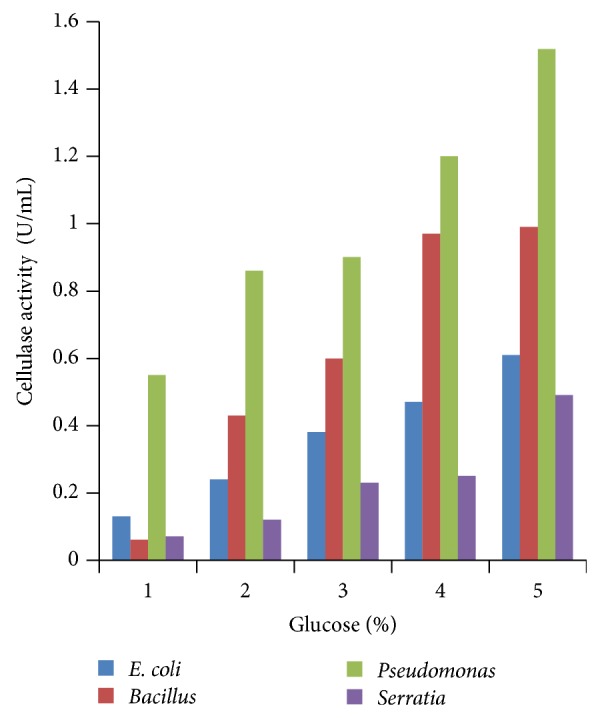
Effect of percent glucose.

**Figure 5:**
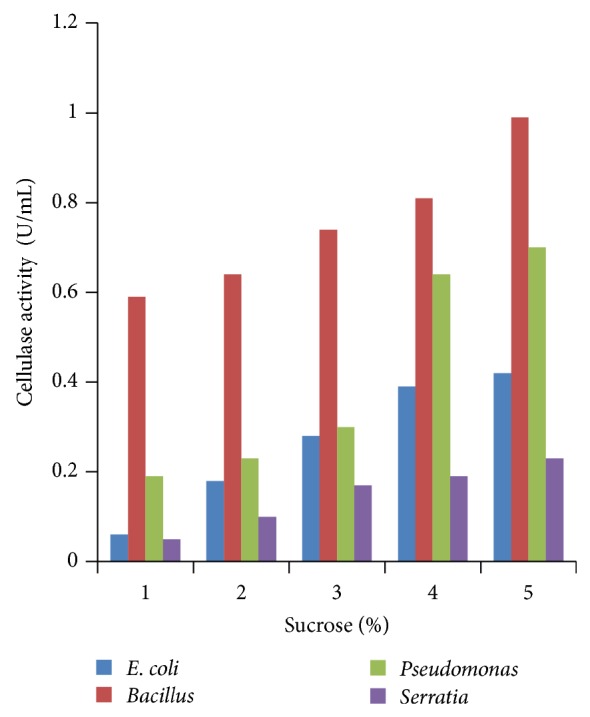
Effect of percent sucrose.

**Figure 6:**
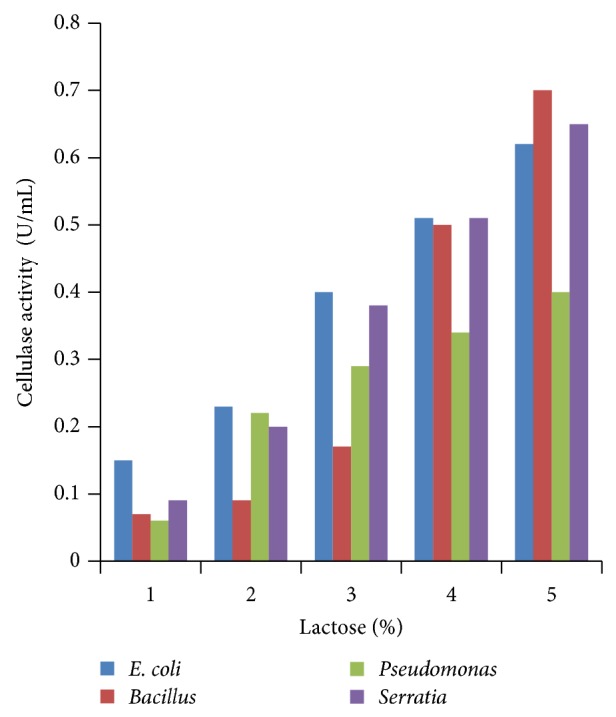
Effect of percent lactose.

**Figure 7:**
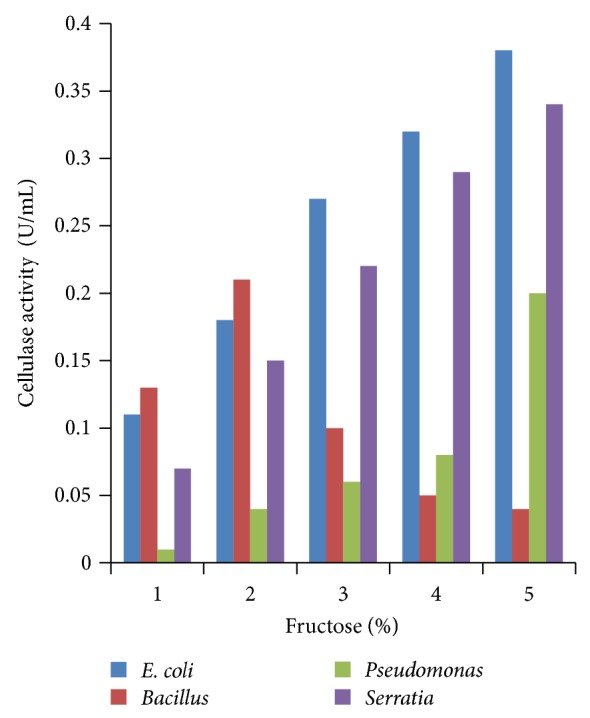
Effect of percent fructose.

**Figure 8:**
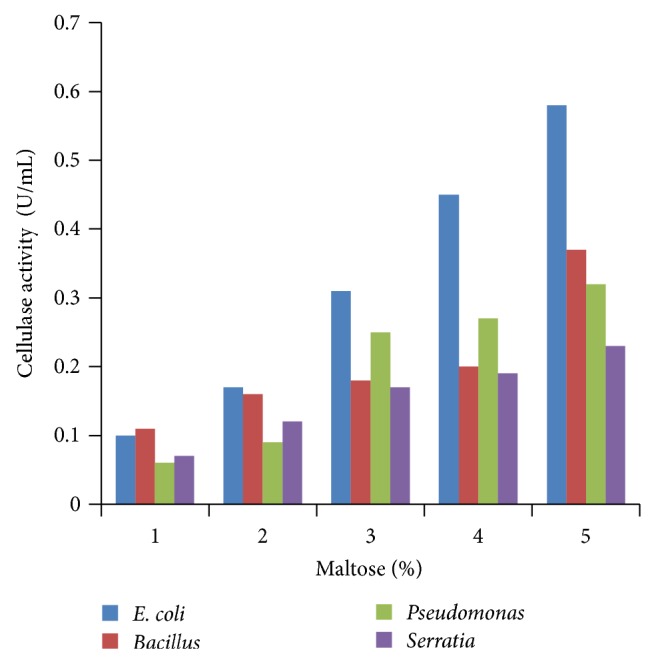
Effect of percent maltose.

Various concentrations of carbon sources were used to replace 1% sugar which was the original concentration in growth media with 2 to 5%. Results obtained showed that 5% carbon source brought the highest cellulase production compared to other % carbon sources at 24 h incubation (Figures 4, 5, 6, 7, and 8). 

### 3.5. Effect of Nitrogen Source

**Figure 9:**
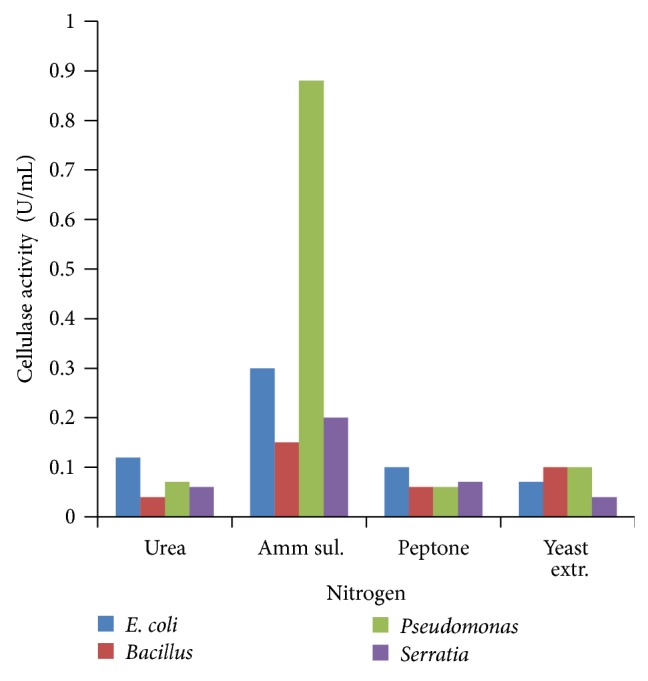
Effect of nitrogen sources.

Production of extracellular cellulase has been shown to be sensitive to repression by different carbohydrate and nitrogen sources. The effect of nitrogen sources was studied in the growth medium, where peptone was replaced by ammonium sulphate, urea, and yeast extract. Among the various nitrogen sources tested, ammonium sulphate was found to be the best nitrogen source for cellulase production (Figure 9). Nitrogen is one of the major cell proteins and stimulation of cellulase activity by ammonium sulphate salt might be due to their direct entry in protein synthesis [29].

### 3.6. Effect of Agro-Based Waste Material

**Figure 10:**
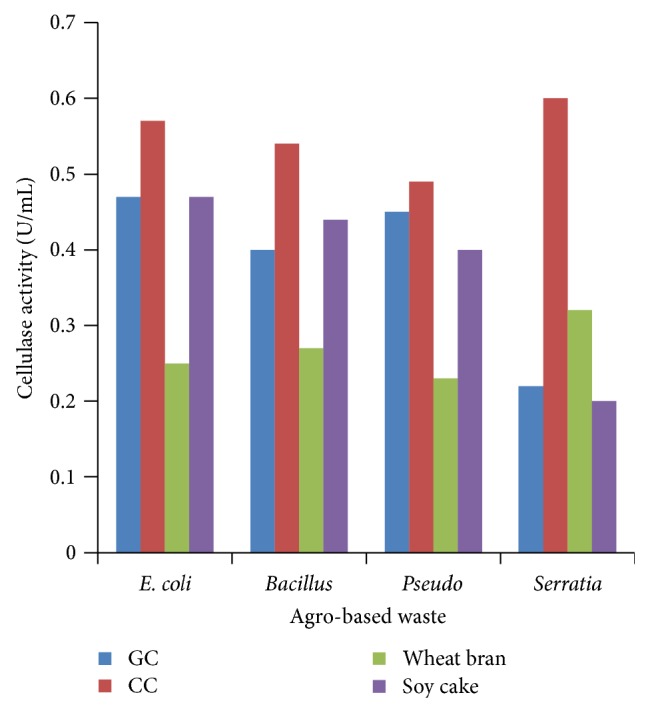
Effect of agro-based waste sources.

The effect of agro based by-products as alternative substrate on bacterial cellulase production under fermentation was studied by several workers. In the present study, coconut cake was found to be the best inducer of cellulase enzyme production by all the four bacterial isolates (Figure 10). 

## 4. Conclusion

The aim of the present work was to isolate and identify a high cellulase producer from soil. *Pseudomonas fulorescens *among* E. coli, Bacillus subtilis, *and* Serratia marscens* produced maximum yield of cellulases. The optimum temperature and pH were determined as 40°C and 9–11 pH and best carbon and nitrogen sources were glucose and ammonium sulphate. This information has enabled the ideal formulation of media composition for maximum cellulase production by this organism. After optimization, the mass production was carried in one litre of optimized media at 40°C for 48 hrs at a pH of 10 on a rotary shaker at 110 rpm. Bacteria, which have high growth rate as compared to fungi, good potential to be used in cellulose production. However, the application of bacteria in producing cellulase is not widely used. 

Cellulolytic property of some bacterial genera such as *Cellulomonas*, *Cellovibrio, Pseudomonas*, *Sporocytophaga *spp. [9], *Bacillus, *and* Micrococcus *[7] was also reported. Enzyme production is closely controlled in microorganisms and for improving its productivity, these controls can be ameliorated. Cellulase yields appear to depend on a complex relationship involving a variety of factors like inoculum size, pH value, temperature, presence of inducers, medium additives, aeration, growth time, and so forth [7].

Further studies were in progress in the purification and application of cellulase in different commercial fields. The purified cellulase can be used for various purposes in detergent industries, food industries, and pharmaceutical industries. The high activity and stability of cellulase enzymes between neutral to alkaline pH and high temperature will be of use in various industrial and biotechnological applications.
